# Prevalence and trends of polypharmacy in U.S. adults, 1999–2018

**DOI:** 10.1186/s41256-023-00311-4

**Published:** 2023-07-12

**Authors:** Xiaowen Wang, Keyang Liu, Kokoro Shirai, Chengyao Tang, Yonghua Hu, Ying Wang, Yuantao Hao, Jia-Yi Dong

**Affiliations:** 1 Center for Public Health and Epidemic Preparedness and Response, Peking University, Beijing, 100191 China; 2 Key Laboratory of Epidemiology of Major Diseases (Peking University), Ministry of Education, Beijing, China; 3 Public Health, Department of Social Medicine, Osaka University Graduate School of Medicine, Osaka, 5650871 Japan; 4 Department of Epidemiology and Biostatistics, School of Public Health, Peking University Health Science Center, Beijing, 100191 China; 5 Medical Informatics Center, Peking University Health Science Center, Beijing, 100191 China; 6 Department of Medical Statistics, School of Public Health, Sun Yat-Sen University, Guangzhou, 510080 China

**Keywords:** Polypharmacy, National Health and Nutrition Examination Survey, NHANES, Trends, Prevalence, Medication

## Abstract

**Background:**

Polypharmacy is one of the most important health issues for its potential impacts on disease burden and healthcare costs. The aim of this study was to update a comprehensive picture of prevalence and trends in polypharmacy over 20 years in U.S. adults.

**Methods:**

Participants included 55,081 adults aged ≥ 20 from the National Health and Nutrition Examination Survey, January 1, 1999, through December 31, 2018. The simultaneously use of ≥ 5 drugs in one individual was defined as polypharmacy. National prevalence and trends in polypharmacy were evaluated among U.S. adults within different demo-socioeconomic status and pre-existing diseases.

**Results:**

From 1999–2000 to 2017–2018, the overall percentages of adults with polypharmacy remained on the rise, increasing from 8.2% (7.2–9.2%) to 17.1% (15.7–18.5%) (average annual percentage change [AAPC] = 2.9%, *P* = .001). The polypharmacy prevalence was considerably higher in the elderly (from 23.5% to 44.1%), in adults with heart disease (from 40.6% to 61.7%), and in adults with diabetes (from 36.3% to 57.7%). Also, we observed a greater increase rate of polypharmacy in men (AAPC = 4.1%, *P* < .001), in the Mexican American (AAPC = 6.3%, *P* < .001), and in the non-Hispanic Black (AAPC = 4.4%, *P* < .001).

**Conclusions:**

From 1999–2000 to 2017–2018, the prevalence of polypharmacy is continually increasing in U.S. adults. The polypharmacy was especially higher in the older, in patients with heart disease, or diabetes. The high prevalence urges the healthcare providers and health policymakers to manage polypharmacy among specific population groups.

**Supplementary Information:**

The online version contains supplementary material available at 10.1186/s41256-023-00311-4.

## Background

Polypharmacy is often commonly defined as the simultaneous use of five or more prescription drugs by one individual. Over the past two decades, it has been observed significant increases in overall prescription drug use and polypharmacy, probably due to large-scale policy changes caused by the introduction of new drugs, research on drug side effects and interactions, or the growing need for treatment of complications [1]. In U.S., it was reported that the prevalence of polypharmacy increased from 8.2% in 1999–2000 to 15% in 2011–2012 [1]. Furthermore, the proportion of polypharmacy among adults aged 65 and older tripled from 12.8% to 39.0% between 1988 and 2010 [2]. A recent meta-analysis reported the pooled polypharmacy prevalence was 37% among individuals over aged 19 [3]. Summarized by another review, the prevalence of polypharmacy varied from 10% to as high as nearly 90% in different populations [4]. Polypharmacy can be a major issue related to prescribed medications, which has become one of the most important but underappreciated health concerns.

Although the use of multiple medications may treat symptoms, prevent disease complications, or increase life expectancy, the majority of research suggests that polypharmacy is associated with negative clinical consequences, including nonadherence to treatment, adverse drug events (e.g., falls, fractures, renal failure), drug-drug interactions, and hospitalizations [5, 6]. Polypharmacy is also linked to increased risks of disability, cognitive decline, and even mortality [7, 8]. In addition, polypharmacy is ordinary among individuals with multimorbidity and an excess of unplanned hospitalizations is seen in those with higher levels of polypharmacy, leading to higher costs of care for individuals and health care systems [9]. Besides, health care tends to be segmented without shared records, as a result, patients with multimorbidity who visit to multiple providers might be prescribed duplicative or interacting treatments [10]. It was estimated that polypharmacy cost at least $50 billion annually from U.S. health plans in 2002, accounting for a large proportion of pharmaceutical expenditure [11].

The substantial increases in prescription medication use and polypharmacy were distinguished particularly in developed countries [12, 13]. However, current guidelines fail to provide adequate information on dosing schedules for people simultaneously using multiple medications, and it was observed that people with polypharmacy may have a higher risk of potentially inappropriate medication use in these developed countries [14]. It is important to have a better understanding of polypharmacy by taking an undated and comprehensive estimation of its prevalence and trend. By using the U.S. as a model case, the aim of this study was to update the national prevalence and trends in polypharmacy among U.S. adults with different demo-socioeconomic status and pre-existing diseases.

## Methods

### Study population

Data for this study were obtained from National Health and Nutrition Examination Survey (NHANES), a study conducted in all 50 states and the District of Columbia by using a complex multi-stage probability sampling method to investigate the health status of the U.S. population. NHANES collected participants’ demographic information, dietary data, examination data, lifestyles, health conditions and biochemical indexes by self-administrated questionnaires, physical examinations, and laboratory tests. All survey participants were eligible.

Data are publicly available at https://www.cdc.gov/nchs/nhanes/index.htm. NHANES has been approved by the Institutional Review Board of the National Center for Health Statistics. All participants have signed informed consent forms.

In this study, ten cycles of 1999–2000, 2001–2002, 2003–2004, 2005–2006, 2007–2008, 2009–2010, 2011–2012, 2013–2014, 2015–2016, and 2017–2018 were included, with a total of 101,316 individuals. We excluded those younger than 20 years, as well as those with incomplete information on polypharmacy. Finally, 55,081 individuals were included in this analysis.

### Assessment of polypharmacy

During the household interview, participants were asked about if they needed any prescriptions by the question: “Have you taken any prescription medicines in the past month?”. Individuals who answered “yes” were asked to show the drug containers for all products used. Meanwhile, the interviewer recorded the number and names of prescription medicines reported.

### Statistical analysis

The prevalence of polypharmacy, defined as simultaneous use of 5 or more drugs, was calculated within every 2-year cycle in NHANES. Survey-weighted regression models were used to estimate the rate of polypharmacy. Since the observed trends might be affected by changes in the age-distribution of the whole population, we also conducted age-adjusted analyses to show the age-standardized polypharmacy rate according to the US 2000 Standard Population (based on 5-year age groups, up to 80 years+). Joinpoint analyses were used to identify points of inflection and calculate the average annual percentage change (AAPC) before and after the inflection points [15].

Subgroup analyses were also conducted to reveal the potential heterogeneity in population with different demo-socioeconomic status and pre-existing diseases. We stratified participants by sex (men and women), age (20–39 years, 40–64 years and ≥  65 years), race (Mexican American, other Hispanic, non-Hispanic White, non-Hispanic Black, and other race—including Multi-Racial), education (high school or below, and college or above), and income (indicated by family income-to-poverty ratio [PIR], where PIR ≤ 1.0 was regarded as poverty). In addition, we also investigated the polypharmacy in participants with specific category of diseases. The self-administrated questionnaires in NHANES provide a broad range of health conditions via in-home personal interviews, including hypertension, high-cholesterol (hypercholesterolemia), diabetes, heart disease (congestive heart failure, coronary heart disease, angina/angina pectoris, or heart attack), respiratory disease (asthma, emphysema, or chronic bronchitis), and cancer. For example, in terms of cancer, participants were asked by the survey questions of “Have you ever been told by a doctor or other health professional that you had had cancer or a malignancy of any kind?”. All analyses were conducted by SAS 9.4 (TS Level 1M6).

## Results

Table 1 shows the prevalence of the participants with polypharmacy in all years, year of 1999–2000, and year of 2017–2018, stratified by population characteristics. During the overall period, a higher prevalence of polypharmacy was observed in women, in the elderly, in the Non-Hispanic White, in adults with education level at high school or below, in adults with heart disease. Similar patterns were observed in the year of 1999–2000 and 2017–2018.

**Table 1:** Prevalence of polypharmacy among adults stratified by population characteristics in overall, year of 1999–2000 and 2017–2018

	Overall (n = 55,081)	1999–2000 (n = 4880)	2017–2018 (n = 5569)
Sex			
Men	4113 (15.5)	210 (9.3)	547 (20.2)
Women	5035 (17.6)	298 (11.4)	581 (20.3)
Age			
20–39 year	407 (2.2)	8 (0.5)	47 (2.8)
40–64 year	3524 (15.9)	188 (10.5)	404 (17.0)
≥ 65 year	5217 (37.1)	312 (22.4)	677 (45.1)
Race			
Mexican American	991 (10.3)	78 (6.1)	95 (12.9)
Other Hispanic	600 (13.3)	31 (10.0)	83 (16.1)
Non-Hispanic White	5007 (20.6)	279 (12.6)	538 (27.8)
Non-Hispanic Black	1975 (17.2)	103 (11.3)	279 (21.5)
Other Race—Including Multi-Racial	575 (11.2)	17 (10.4)	133 (12.3)
Education level			
High School or below	5132 (18.4)	339 (11.3)	559 (22.9)
College or above	3993 (14.7)	163 (8.8)	566 (18.2)
Family income-to-poverty ratio (PIR)			
≤ 1.0	2735 (17.4)	188 (11.6)	338 (19.9)
> 1.0	6413 (16.3)	320 (9.8)	790 (20.4)
Disease status			
Hypertension	6822 (35.7)	347 (23.3)	862 (40.7)
High-cholesterol	5489 (32.5)	243 (20.8)	716 (36.8)
Diabetes	3673 (55.0)	184 (38.3)	523 (59.7)
Heart disease	2944 (59.1)	190 (42.1)	346 (65.7)
Respiratory disease	2854 (30.1)	161 (21.9)	371 (34.3)
Cancer	1843 (35.7)	76 (19.7)	243 (41.3)

Values are presented as unweighted frequency (percentage)

**Fig. 1:**
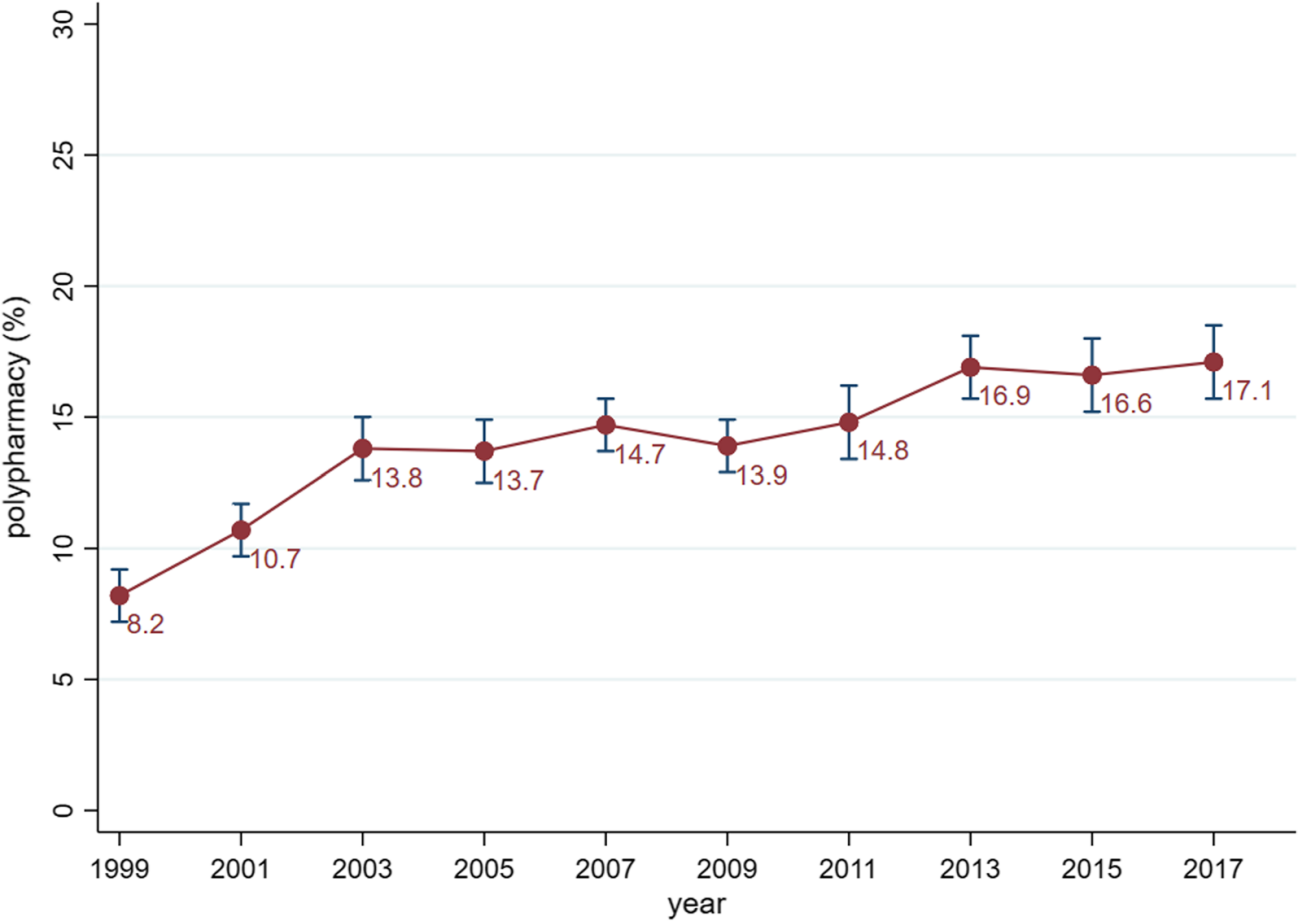
Prevalence and trend of polypharmacy among U.S. adults (1999–2018)

**Fig. 2:**
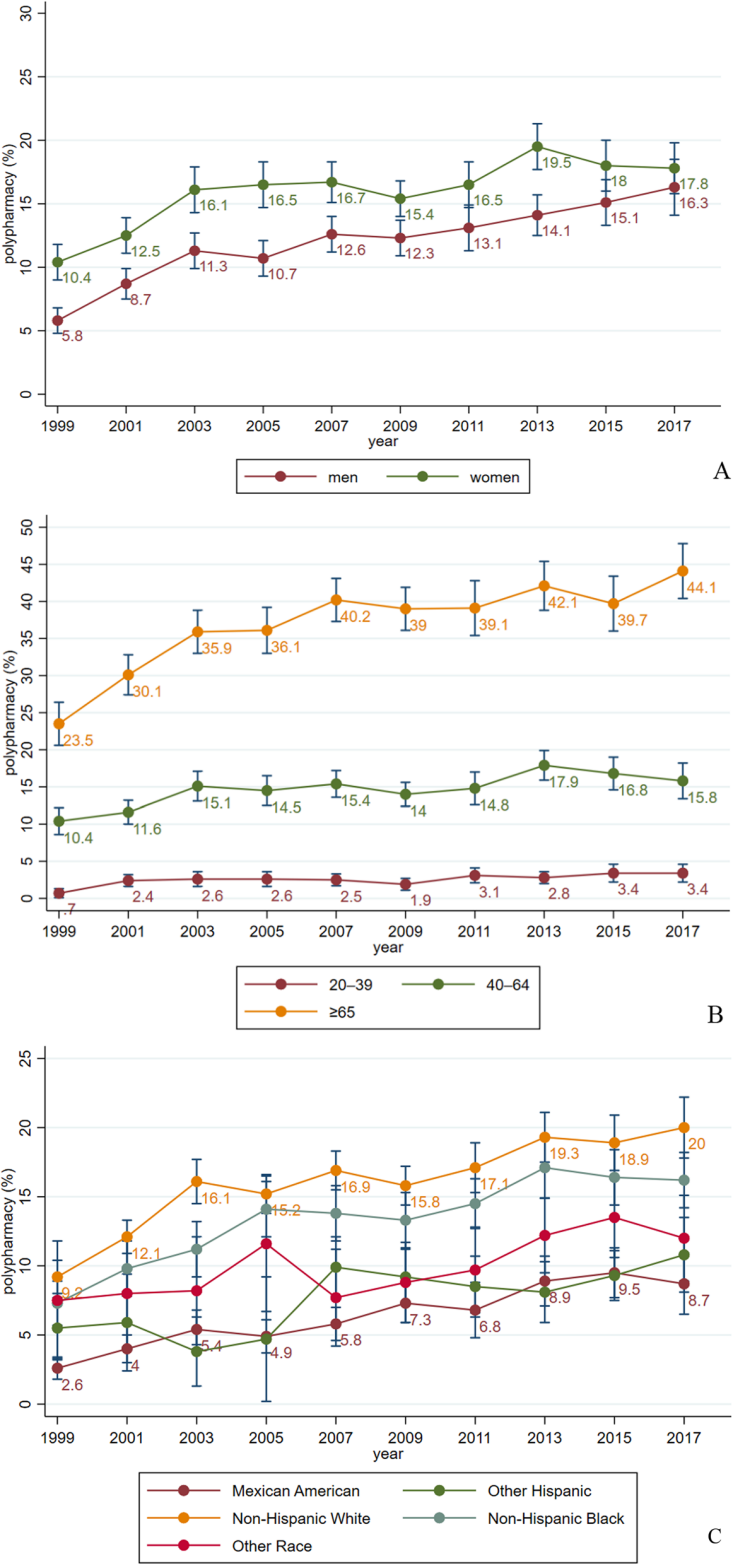
Prevalence and trend of polypharmacy among U.S. adults, by sex (**A**), age group (**B**), and race (**C**) (1999–2018)

From 1999–2000 to 2017–2018, the overall percentages of adults with polypharmacy remained on the rise, increasing from 8.2% (95% CI 7.2 to 9.2) to 17.1% (95% CI 15.7 to 18.5) (AAPC = 2.9%, *P* = 0.001, Fig. 1). This pattern remained unchanged with age-adjustment model (Additional file 1: Fig. S2). Furthermore, similar trends were observed in subgroup analyses. In the sex-stratified analysis, the prevalence of polypharmacy was consistently higher in women than in men. From 1999–2000 to 2017–2018, the prevalence of polypharmacy increased from 10.4% (95% CI 9.0 to 11.8) to 17.8% (95% CI 15.8 to 19.8) and increased from 5.8% (95% CI 4.8 to 6.8) to 16.3% (95% CI 14.1 to 18.5) in women and men, respectively (Fig. 2A). However, the AAPC was relatively higher in men (4.1%, *P* < 0.001) than that in women (2.4%, *P* = 0.006). In the age-stratified analysis, the polypharmacy prevalence was consistently higher in participants aged 65 years and older than that in the 40–64 years group and simultaneously higher than in the 20–39 years group. Between 1999–2000 and 2017–2018, the prevalence increased from 23.5% (95% CI 20.6 to 26.4) to 44.1% (95% CI 40.4 to 47.8) in the 65 years and older group, while it increased from 10.4% (95% CI 8.6 to 12.2) to 15.8% (95% CI 13.4 to 18.2) and from 0.7% (95% CI 0.1 to 1.3) to 3.4% (95% CI 2.2 to 4.6) in the 40–64 years and 20–39 years groups, respectively (Fig. 2B). The AAPCs ranged between 2.1 to 2.9 and did not differ much across age groups. In the race-stratified analysis, the non-Hispanic White consistently had the highest prevalence during the observation period, rising from 9.2% (95% CI 8.0 to 10.4) to 20.0% (95% CI 17.8 to 22.2). This was followed by the non-Hispanic Black, which increased from 7.3% (95% CI 5.7 to 8.9) to 16.2% (95% CI 14.2 to 18.2). Mexican Americans had the lowest prevalence rate, with the prevalence rising from 2.6% (95% CI 1.8 to 3.4) to 8.7% (95% CI 6.5 to 10.9) (Fig. 2C). However, the AAPCs were higher in the Mexican American (6.3%, *P* < 0.001) and in the non-Hispanic Black (4.4%, *P* < 0.001) than that in other races.

**Fig. 3:**
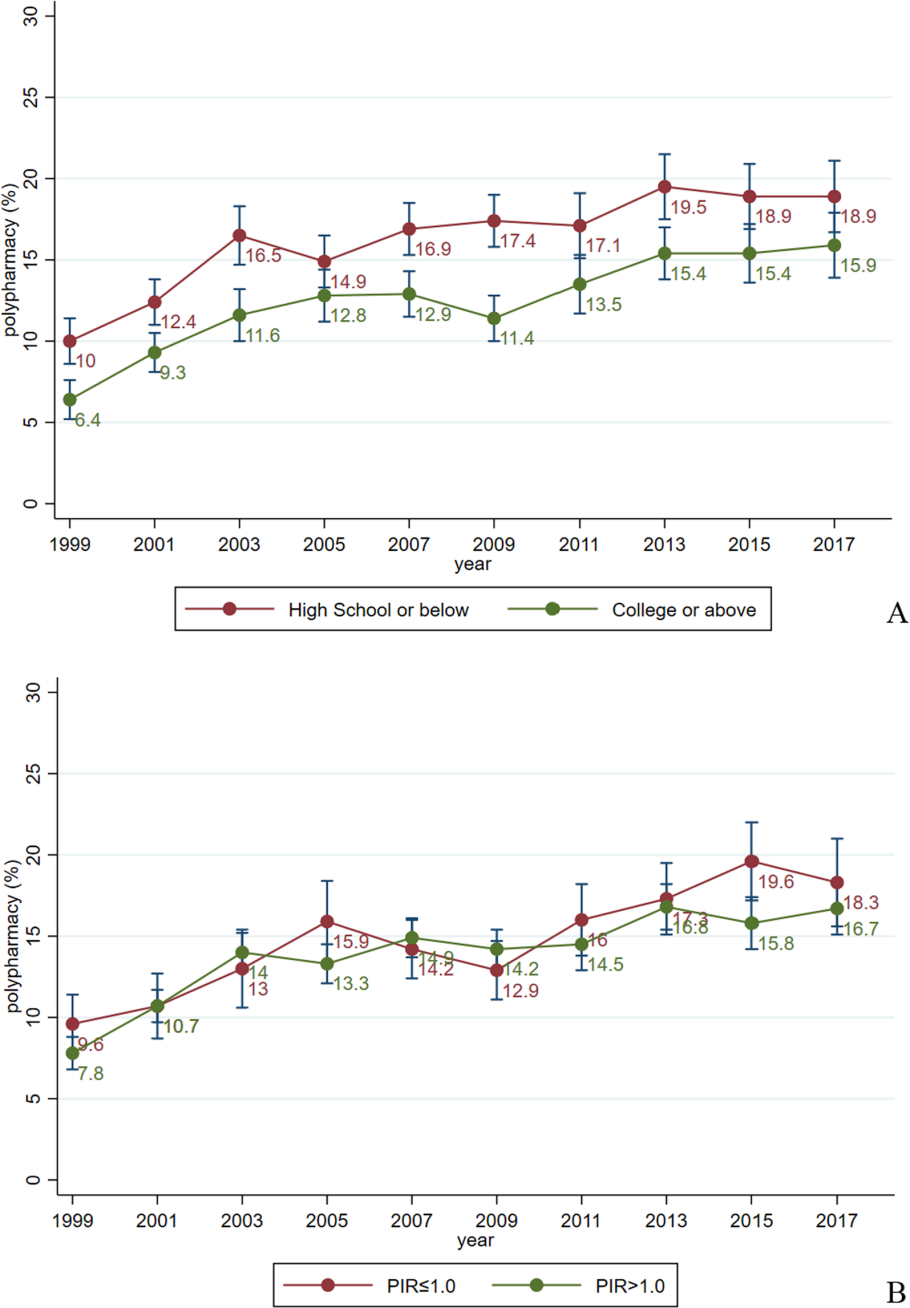
Prevalence and trend of polypharmacy among U.S. adults, by education (**A**), and by income (**B**) (1999–2018)

Grouped by different levels of education, the prevalence of polypharmacy was consistently higher in adults with low education (high school or below) than in those with high education (college or above). Among adults with education at the high school level or below, the polypharmacy prevalence increased from 10.0% (95% CI 8.6 to 11.4) to 18.9% (95% CI 16.7 to 21.1) from 1999–2000 to 2017–2018, while among those with education at the university level or above it increased from 6.4% (95% CI 5.2 to 7.6) to 15.9% (95% CI 13.9 to 17.9) (Fig. 3A). As for the level of poverty, there was no apparent difference in polypharmacy prevalence between populations below or above the poverty level (Fig. 3B).

**Fig. 4:**
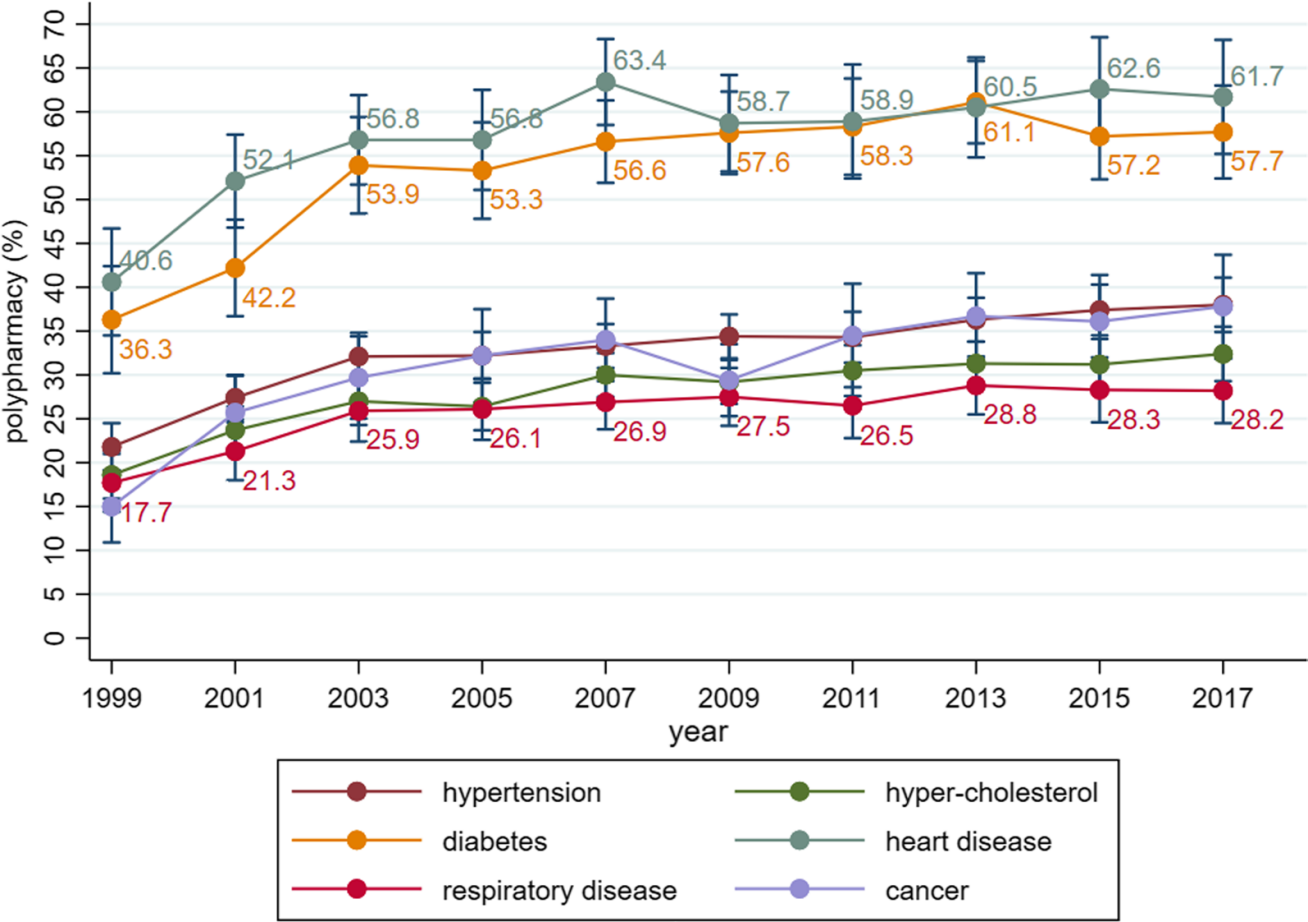
Prevalence and trend of polypharmacy among U.S. adults, by diagnosed disease (1999–2018)

In addition, according to participants self-reporting whether they had a certain disease diagnosed by a doctor, we divided the participants into six groups by suffering from hypertension, high-cholesterol, diabetes, heart disease, respiratory diseases, and cancer. From 1999–2000 to 2017–2018, it was observed that patients with heart disease had the highest prevalence of polypharmacy, rising from 40.6% (95% CI 34.5 to 46.7) to 61.7% (95% CI 55.2 to 68.2). The prevalence of polypharmacy was also considerably high among adults with diabetes, which rose from 36.3% (95% CI 30.2 to 42.4) to 57.7% (95% CI 52.4 to 63.0). The polypharmacy prevalence among those with high-cholesterol and respiratory diseases was relatively low. Among adults with respiratory diseases, the prevalence increased from 17.7% (95% CI 14.4 to 21.0) to 28.2% (95% CI 24.5 to 31.9) (Fig. 4). The AAPCs ranged between 1.2% (heart disease) to 2.7% (cancer).

## Discussion

From 1999–2000 to 2017–2018, there was an overall increasing trend in the prevalence of polypharmacy in U.S. adults, from 8.2% to 17.1%, with an AAPC of 2.9%. The polypharmacy prevalence was considerably higher in the elderly, in adults with heart disease, and in adults with diabetes. Also, we observed a greater increase rate of polypharmacy in men, in the Mexican American, and in the non-Hispanic Black.

The prevalence of polypharmacy has increased over the past several decades, during which several clinical guidelines such as Beers Criteria [16], the STOPP/START criteria [17], and the European Union SIMPATHY project [18] have been published for safe medication use and polypharmacy management. In this present study, we found the large increases in 1999–2003 that then plateaued, particularly for the older adults, which might be partially explained by the publication of the updated Beers list for older adults in 2003. A previous study also supported that the proportion of persons on a potentially inappropriate medication has decreased since the updated Beers criteria in 2003 [2]. Nevertheless, the highest polypharmacy was still detected in the elder population, with reported prevalence ranging from 26 to 44% [19–21]. Particularly, in older adults with frailty, the overall prevalence of polypharmacy and hyper-polypharmacy (the concurrent use of ten or more medications) was 59% and 22%, respectively [22]. Compared to people without polypharmacy, polypharmacy was inclined to accelerate frailty states progression, leading to adverse clinical outcomes and a higher risk of mortality in older patients with frailty [22]. Our recent study also found that polypharmacy was associated with increased risks of all-cause and cardiovascular mortality among the elderly chronic kidney disease patients [8]. In real-world clinical settings, the majority of older adults suffered from multimorbidity and polypharmacy, though the use of multiple medications may treat symptoms effectively for multimorbidity, it is crucial to explore the clinical trajectories of individuals by focusing on the dynamics and complexities of multimorbidity and polypharmacy [23, 24].

Consistent with the literature, this research found that women were more likely to have polypharmacy than men [23, 25]. This may be partially interpreted by the longer life expectancy and longer years with multimorbidity in women. Also, women are more likely to be influenced by psychiatric and social factors, leading to higher symptoms perception and health-seeking behaviors [26, 27]. However, a greater increase rate of polypharmacy in men needs further attention. In addition, previous research has established that there exist racial and ethnic disparities in prescription drug use. For example, data from the Medical Expenditure Panel Survey showed non-Hispanic whites were more likely to have access to new medications than non-Hispanic blacks and Hispanic whites [28], which was similar to our results that polypharmacy reached the highest level in non-Hispanic whites. Factors associated with racial disparities in medication use have been explored in several studies. Minorities might be less inclined to attempt new drugs, delay health-seeking behaviors, or enroll fewer health care services [29].

This present study found the highest prevalence of polypharmacy in individuals with heart disease. Heart disease is the leading cause of disability and death in the U.S., and its prevalence is still on the rise [30, 31]. Recent guidelines for the treatment of cardiovascular disease recommend the use of multiple medications to prevent complications or reduce mortality [32]. However, this is also accompanied by potential problems such as poor adherence, adverse drug reactions, hospitalizations, higher mortality burden, and substantial healthcare cost [33]. A prior study reported polypharmacy prevalence was high among people with heart failure, ranging from 17.2% to 99% [34], as well as among people with type 2 diabetes, varying from 57 to 99% [35]. Many recommendations to manage polypharmacy in people with cardiovascular diseases or diabetes have been gradually proposed in order to strike a balance between unnecessary medications and effective treatments [36, 37]. For example, patients with heart disease could have many comorbid conditions, which could be a main cause of high polypharmacy, therefore, certain particular drug class could be prioritized to treat multiple conditions [37]. Besides, new support tools have been gradually developed for patients with chronic disease to support physicians in deprescribing [38, 39].

By using nationally representative data, we provided an update prevalence and trend of polypharmacy over a 20-year period in the U.S. adults. The subgroup analyses also offered a more comprehensive picture to investigate the polypharmacy across people subgroups including sex, age, race, socioeconomic status, and pre-existing disease. Furthermore, the medication information was collected by using standard methods, and the interviewer could check the medication containers during the household interview. Data were also routinely examined for quality assurance and quality control. However, several limitations should be addressed. First, although we used sampling weights to calculate the prevalence, the non-response bias could still occur. Second, we only accessed the number of drug prescription, but the specific categories of drugs and the duration of drug use were not analyzed. Besides, data from NHANES failed to include prescriptions over the counter or herbal supplements and pro re nata (PRN) medications, potentially contributing to an underestimation of polypharmacy rates. Finally, the diseases were self-reported by participants, which might result in measurement errors and misclassification. However, previous studies have proved high validity of clinical records in NHANES [40, 41].

## Conclusions

From 1999–2000 to 2017–2018, the prevalence of polypharmacy was continually increasing in U.S. adults. The polypharmacy was especially higher in the elder, in patients with heart disease, and in patients with diabetes. The high prevalence urges the healthcare providers and health policymakers to develop and implement measures targeted at polypharmacy among specific population groups, which might be of great potential significance to the delivery of appropriate and safe medication.

## Supplementary Information

**Additional file 1: Fig. S1**. Results of Joinpoint trend analysis for polypharmacy among U.S. adults, 1999-2018. **Fig. S2**. Results of age-adjusted* Joinpoint trend analysis for polypharmacy among U.S. adults, 1999-2018. **Table S1**. The average annual percent change (AAPC) estimates based on Joinpoint Analyses for polypharmacy among U.S. adults, 1999-2018

## Data Availability

The data involved in this study are freely available from https://www.cdc.gov/nchs/nhanes/index.htm.

## Abbreviations

AAPC: Average annual percentage change
NHANES: National Health and Nutrition Examination Survey
