# Supplementary material for: Prevalence and trends of polypharmacy in U.S. adults, 1999–2018
Source: Glob Health Res Policy. 2023 Jul 12;8:25. doi: 10.1186/s41256-023-00311-4 (PMC10337167; doi:10.1186/s41256-023-00311-4)
Supplement: Supplementary file 1 — Additional file 1: Fig. S1. Results of Joinpoint trend analysis for polypharmacy among U.S. adults, 1999-2018. Fig. S2. Results of age-adjusted* Joinpoint trend analysis for polypharmacy among U.S. adults, 1999-2018. Table S1. The average annual percent change (AAPC) estimates based on Joinpoint Analyses for polypharmacy among U.S. adults, 1999-2018 [file 41256_2023_311_MOESM1_ESM.docx]

Supplementary Figure S1. Results of Joinpoint trend analysis for polypharmacy among U.S. adults, 1999-2018.

Supplementary Figure S2. Results of age-adjusted* Joinpoint trend analysis for polypharmacy among U.S. adults, 1999-2018.

Supplementary Table S1. The average annual percent change (AAPC) estimates based on Joinpoint Analyses for polypharmacy among U.S. adults, 1999-2018


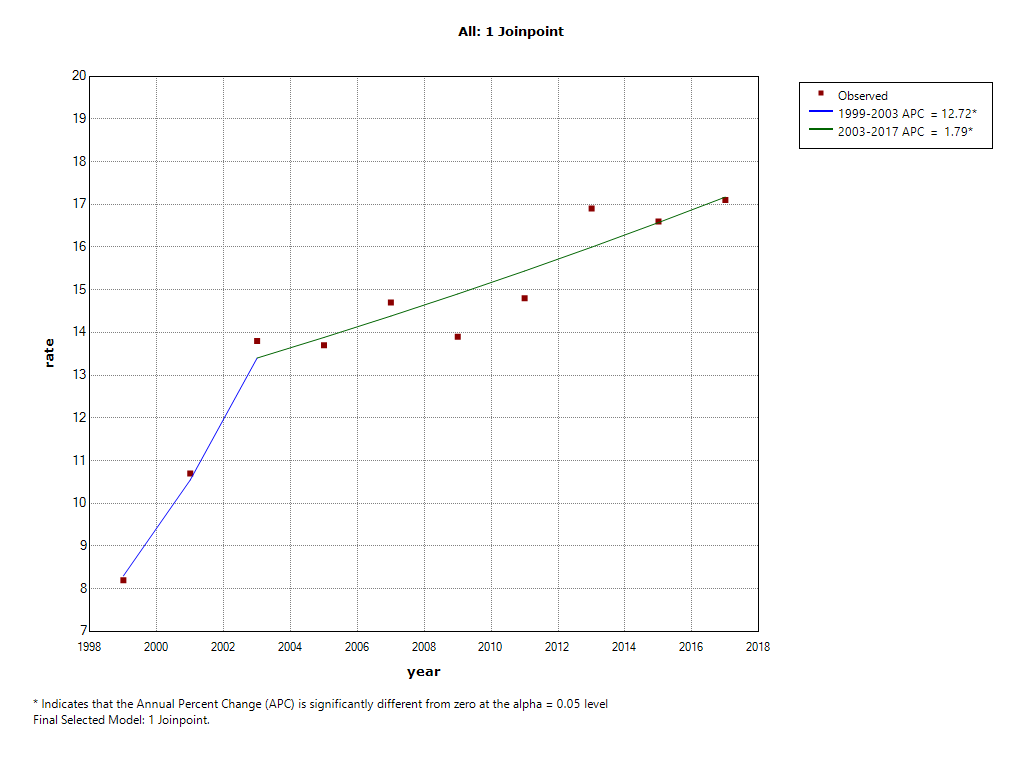
 Supplementary Figure S1. Results of Joinpoint trend analysis for polypharmacy among U.S. adults, 1999-2018.


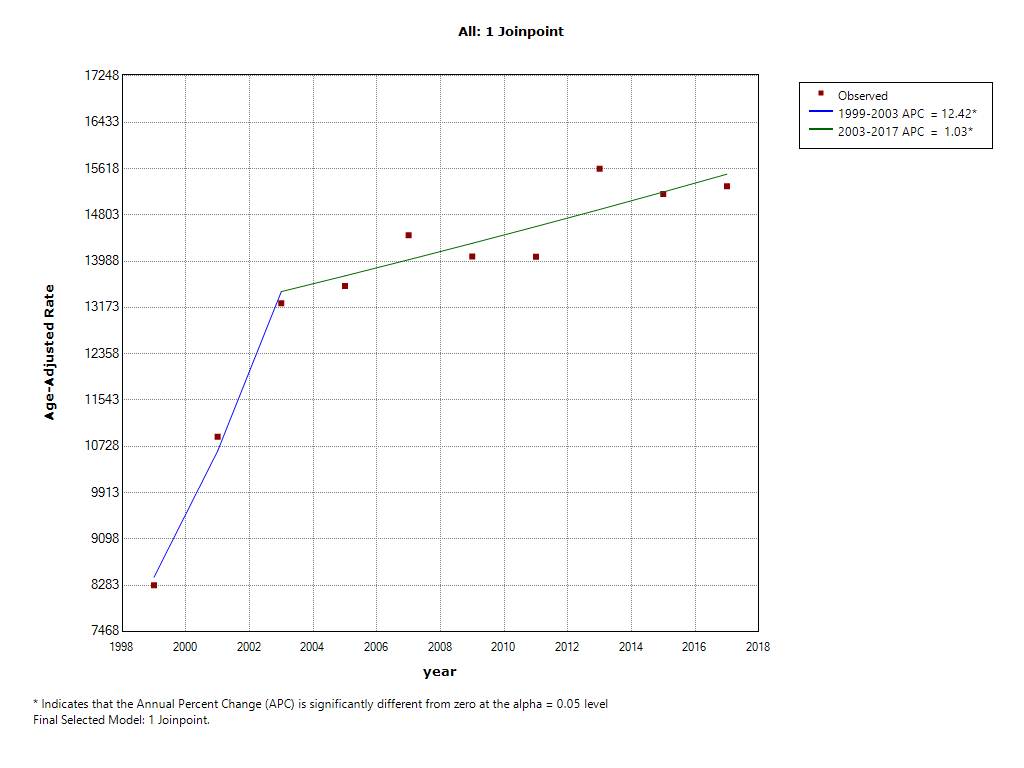


Supplementary Figure S2 Results of age-adjusted* Joinpoint trend analysis for polypharmacy among U.S. adults, 1999-2018.

* Age-adjusted using standardization and the 2000 US Standard Population (based on 5-year age groups, up to 80y+).

Supplementary Table S1. The average annual percent change (AAPC) estimates based on Joinpoint Analyses for polypharmacy among U.S. adults, 1999-2018

|  | AAPC | Lower CI | Upper CI | Test statistic^+^ | P-value |
| --- | --- | --- | --- | --- | --- |
| Total | 2.9* | 1.6 | 4.4 | 4.9 | 0.001 |
| Total (Standardized) | 2.3* | 0.9 | 3.7 | 3.9 | 0.005 |
| Sex |  |  |  |  |  |
| Men | 4.1* | 2.5 | 5.8 | 5.8 | <0.001 |
| Women | 2.4* | 0.9 | 3.9 | 3.7 | 0.006 |
| Age |  |  |  |  |  |
| 20–39 yr | 2.9 | -0.0 | 5.9 | 2.3 | 0.051 |
| 40–64 yr | 2.1* | 0.8 | 3.5 | 3.6 | 0.007 |
| ≥65 yr | 2.3* | 1.0 | 3.5 | 4.2 | 0.003 |
| Race |  |  |  |  |  |
| Mexican American | 6.3* | 4.4 | 8.2 | 7.8 | <0.001 |
| Other Hispanic | 2.7* | 0.1 | 5.5 | 2.4 | 0.044 |
| Non-Hispanic White | 3.2* | 1.7 | 4.8 | 4.8 | 0.001 |
| Non-Hispanic Black | 4.4* | 2.1 | 6.9 | 3.7 | <0.001 |
| Other Race | 3.0 | -1.7 | 7.9 | 1.2 | 0.219 |
| Education |  |  |  |  |  |
| High School or below | 2.8* | 1.4 | 4.3 | 4.6 | 0.002 |
| College or above | 3.6* | 1.9 | 5.3 | 5.0 | 0.001 |
| Family income-to-poverty ratio (PIR) |  |  |  |  |  |
| ≤1.0 | 3.5 | 2.1 | 4.9 | 5.8 | <0.001 |
| >1.0 | 2.9* | 1.2 | 4.5 | 4.1 | 0.004 |
| Disease |  |  |  |  |  |
| Hypertension | 2.1* | 1.2 | 3.0 | 5.2 | 0.001 |
| Hyper-cholesterol | 2.1 | 1.1 | 3.1 | 4.8 | 0.001 |
| Diabetes | 1.6* | 0.3 | 2.9 | 2.9 | 0.019 |
| Heart disease | 1.2* | 0.2 | 2.3 | 2.7 | 0.029 |
| Respiratory disease | 1.7* | 0.6 | 2.9 | 3.5 | 0.008 |
| Cancer | 2.7* | 0.9 | 4.5 | 3.5 | 0.008 |

*Indicates that the AAPC is significantly different from zero at the alpha=0.05 level.

^+^ If the AAPC is within one segment, the t-distribution is used. Otherwise, the normal (z) distribution is used.
